# A Novel Predictive Tool for Determining the Risk of Early Death From Stage IV Endometrial Carcinoma: A Large Cohort Study

**DOI:** 10.3389/fonc.2020.620240

**Published:** 2020-12-14

**Authors:** Zixuan Song, Yizi Wang, Yangzi Zhou, Dandan Zhang

**Affiliations:** Department of Obstetrics and Gynecology, Shengjing Hospital of China Medical University, Shenyang, China

**Keywords:** endometrial carcinoma, early death, nomograms, SEER database, prognosis

## Abstract

**Background:**

Endometrial carcinoma is a common gynecological malignancy. Stage IV endometrial carcinoma is associated with a high risk of early death; however, there is currently no effective prognostic tool to predict early death in stage IV endometrial cancer.

**Methods:**

Surveillance, Epidemiology, and End Results (SEER) data from patients with stage IV endometrial cancer registered between 2004 and 2015 were used in this study. Important independent prognostic factors were identified by univariate and multivariate logistic regression analyses. A nomogram of all-cause and cancer-specific early deaths was constructed using relevant risk factors such as tumor size, histological grade, histological classification, and treatment (surgery, radiotherapy, chemotherapy).

**Results:**

A total of 2,040 patients with stage IV endometrial carcinoma were included in this study. Of these, 299 patients experienced early death (≤3 months) and 282 died from cancer-specific causes. The nomogram of all-cause and cancer-specific early deaths showed good predictive power and clinical practicality with respect to the area under the receiver operating characteristic curve and decision curve analysis. The internal validation of the nomogram revealed a good agreement between predicted early death and actual early death.

**Conclusions:**

We developed a clinically useful nomogram to predict early mortality from stage IV endometrial carcinoma using data from a large cohort. This tool can help clinicians screen high-risk patients and implement individualized treatment regimens.

## Introduction

Endometrial carcinoma is the fourth most common gynecological malignancy in developed countries and the sixth leading cause of cancer death among women (1). The incidence of endometrial carcinoma is increasing, mainly due to factors such as obesity, low fertility, and the use of non-antagonistic estrogen (2, 3). According to histology, endometrial carcinoma can be divided into two subtypes (4). Subtype 1 (endometrioid endometrial carcinoma) accounts for approximately 80% of endometrial cancers; it is easily diagnosable in early stages and has a good prognosis. Subtype 2 (non-endometrioid endometrial carcinoma) accounts for approximately 20% of endometrial carcinomas, is highly invasive, and has a poor prognosis (5). Most patients with endometrial carcinoma are diagnosed at an early stage of the disease, and the 5-year survival rate is relatively high (65.27%–81.2%) (6, 7). However, stage IV patients, though few in number, have a poor survival outcome (8). In a study by Tai et al., the survival of patients with stage IV endometrial cancer was found to be significantly poor, with the median progression-free survival and overall survival being 3.8 and 12.3 months, respectively (9). In fact, endometrial cancer can sometimes be diagnosed at its onset with localization to the distal bones, and that the treatment of this subset of patients is not well defined (10, 11).

However, patients with stage IV endometrial carcinoma are still prone to early death, the cause of which is yet to be investigated (6). Exploration of factors associated with early death can help clinicians identify patients at high risk of early death and develop targeted treatment plans to improve patient survival and quality of life. However, to our knowledge, no study has thoroughly investigated mortality within 3 months from diagnosis due to endometrial carcinoma. Therefore, an early death prediction model for endometrial carcinoma is required, so as to guide gynecological oncologists to conduct individualized treatment for patients.

Nomograms are widely used to predict the incidence and prognosis of diseases, and in recent years, gynecologists have also been focusing on this tool (12, 13). For endometrial carcinoma, only a few studies have utilized nomograms for visual prediction models, and most of these studies are based on cohorts with a small sample size, which may reduce the stability and universality of the nomograms (14–17).

The Linked Surveillance, Epidemiology, and End Results (SEER) database (https://seer.cancer.gov/) has collected tumor incidence and survival data of about 34.6% of the United States population cancer registry, which is the authoritative source of tumor information in the United States (18). Studies based on the SEER database can target different regions and larger populations than single-center studies. In this study, a SEER database-based cohort of patients with stage IV endometrial carcinoma was used to assess the factors associated with early death (survival duration ≤3 months) in order to construct a predictive model for its incidence in cases of endometrial carcinoma.

## Materials and Methods

### Ethics Statement

The SEER database data does not need informed patient consent, and cancer is a reportable disease in every state in the United States. This study was consistent with the 1964 Helsinki Declaration and subsequent amendments or similar ethical standards.

### Patients

**Figure 1:**
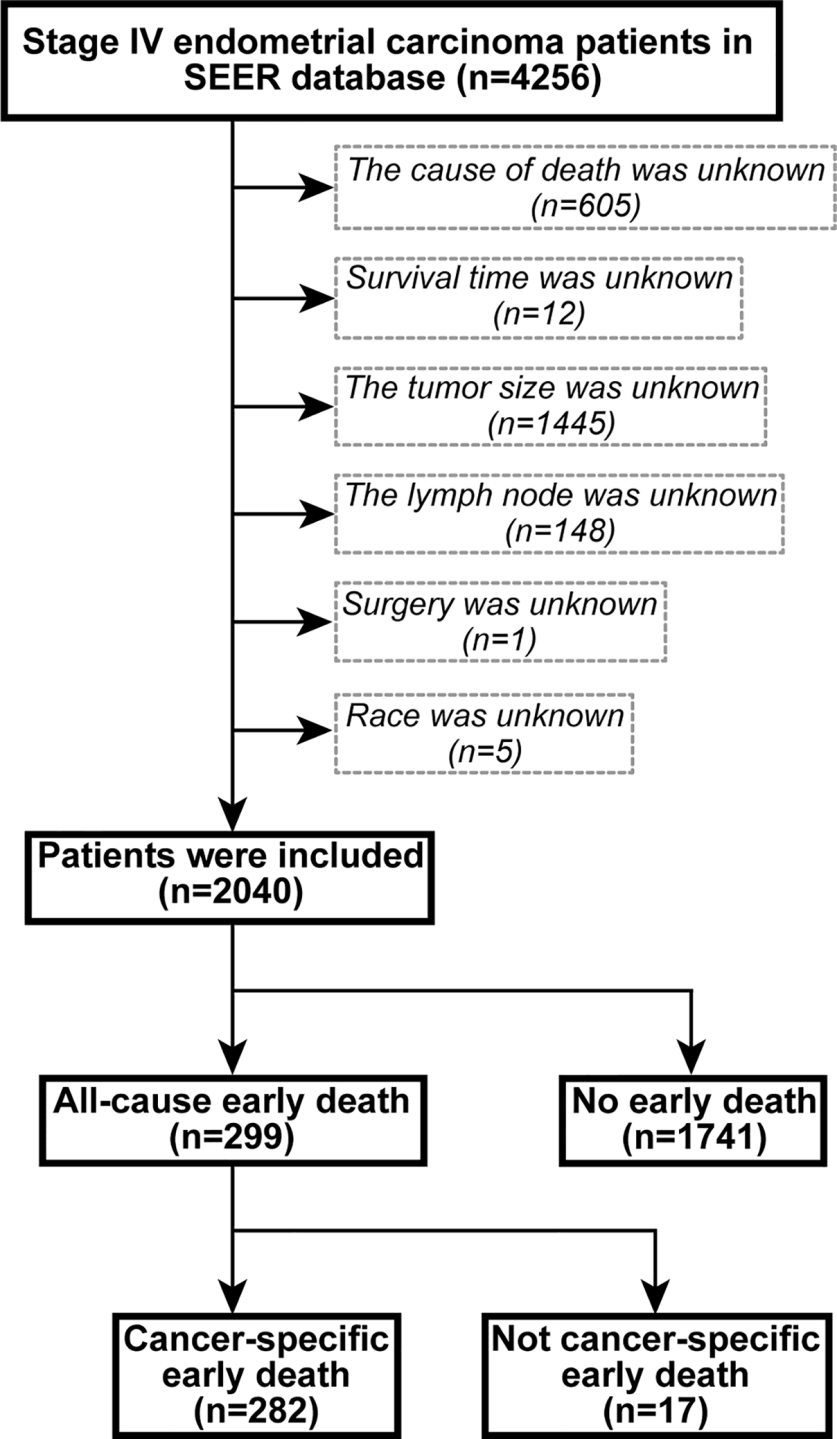
The flowchart of patient selection.

Data from patients with the International Federation of Gynecology and Obstetrics (FIGO) stage IV endometrial carcinoma registered from 2004 to 2015 were extracted using SEER*Stat version 8.3.6.1. The inclusion site codes were c54.0-C54.3, C54.8, C54.9, and C55.9, and the histological codes were 8140/3-8384/4, according to the International Classification of Tumor Diseases, Third Edition (ICD-O-3). The exclusion criteria were (1) unknown cause of death, (2) unknown duration of survival, (3) unknown tumor size, (4) unknown regional lymph node information, (5) unknown surgical treatment, and (6) unknown ethnicity. The flow chart of the patient selection criteria is shown in **Figure 1**. Based on previous studies, early death was defined as death within 3 months from the initial diagnosis (19, 20).

### Data Collection

Demographic and clinical characteristics of endometrial carcinoma patients were extracted from the SEER database. These characteristics included diagnostic age; race; insurance status; marital status; tumor size; histological grade; regional lymph node status; presence of bone, brain, liver, and lung metastases; and mode of treatment (surgery, radiotherapy, and chemotherapy). The primary outcomes of this study were all-cause early death and cancer-specific early death.

### Statistical Analysis

**Figure 2:**
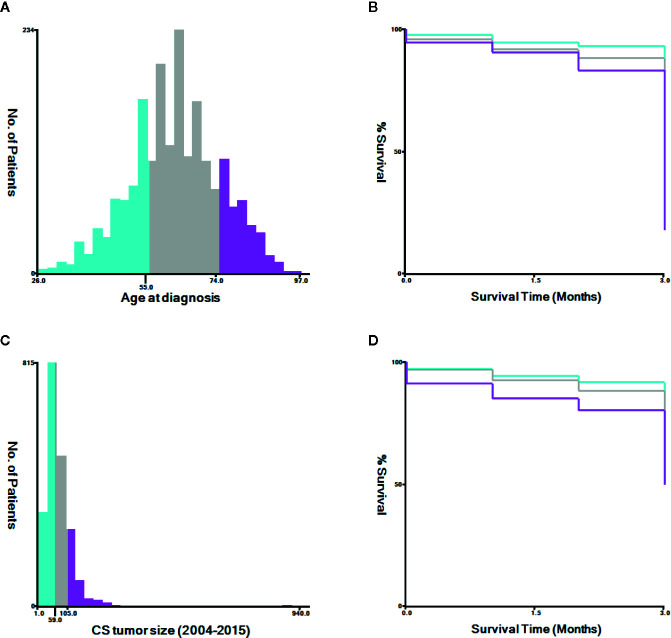
The appropriate cutoff values of age and tumor size was assessed by X-tile analysis. **(A, B)** The appropriate cutoff values of age were 55 and 74 years. **(C, D)** The appropriate cutoff values of tumor size were 59 and 105 mm.

Appropriate cutoff values for age and tumor size were assessed using the X-tile software (Yale University, New Haven, Connecticut, USA) (21) (**Figure 2**). The appropriate cutoff values for age were 55 and 74 years and those for tumor size were 59 and 105 mm, respectively. R-version 3.6.3 (R Foundation for Statistical Computing, Vienna, Austria, http://www.r-project.org) was used to analyze all data in the RStudio environment. P < 0.05 was considered statistically significant. Univariate and multivariate logistic regression analyses were performed using clinical data to assess factors associated with early mortality. Odds ratios (ORs) and 95% confidence intervals (CIs) were calculated. We used the R language rms package to predict early death by developing nomograms based on associated risk factors among patients with uterine endometrial cancer. We used the R language pROC package for the receiver operating characteristic (ROC) curve. Nomograms were evaluated by studying the area under the ROC curve (AUC) (22). We used the DCA package in R language to evaluate the clinical effects of the nomogram through decision curve analysis (DCA) (23) and calculated the net benefit under each risk threshold probability. The k-fold cross-validation (K=10) was used for internal validation, and the concordance statistic (C-statistic) (24) and Brier score (25) comparing the original data and the validation model were used to evaluate the accuracy of the nomogram.

## Results

### Patient Characteristics

Of 4256 SEER database patients with endometrial carcinoma, 2,040 were included in the study based on the aforementioned inclusion and exclusion criteria. Of these, 299 patients died early from all causes, and 282 died early from cancer-specific causes. The majority of early deaths occurred in patients who were white (75%), between the ages of 56 and 74 years (55%), and insured (72%). The most common histological grades among patients who died early were III–IV (66%), and the most common histological classification was non-endometrioid endometrial carcinoma (51%). Most patients who died early had no bone (47%), brain (54%), liver (43%), or lung metastases (31%). Moreover, most of them were treated surgically (57%), without radiation therapy (93%), and without chemotherapy (78%). The characteristics of the patients are shown in **Table 1**.

**Table 1:** Characteristics with stage IV endometrial carcinoma patients.

Characteristic	No early death N=1741	All-cause early death N=299	Cancer-specificearly death N=282
**Age**			
≤55	498 (29%)	48 (16%)	48 (17%)
56–74	968 (56%)	164 (55%)	153 (54%)
≥75	275 (16%)	87 (29%)	81 (29%)
**Race**			
White patients	1,330 (76%)	223 (75%)	213 (76%)
African American	223 (13%)	41 (14%)	36 (13%)
Others*	188 (11%)	35 (12%)	33 (12%)
**Insurance**			
Uninsured	61 (3.5%)	16 (5.4%)	16 (5.7%)
Insured	1,356 (78%)	214 (72%)	200 (71%)
Unknown	324 (19%)	69 (23%)	66 (23%)
**Marital status**			
Unmarried	395 (23%)	62 (21%)	57 (20%)
Married	790 (45%)	110 (37%)	104 (37%)
Divorced or Separated	215 (12%)	35 (12%)	35 (12%)
Widowed	274 (16%)	84 (28%)	78 (28%)
Unknown	67 (3.8%)	8 (2.7%)	8 (2.8%)
**Tumor size(mm)**			
≤59	814 (47%)	95 (32%)	93 (33%)
60-105	695 (40%)	126 (42%)	116 (41%)
≥106	232 (13%)	78 (26%)	73 (26%)
**Histology grade**			
GI-II	492 (28%)	38 (13%)	34 (12%)
GIII-IV	956 (55%)	198 (66%)	188 (67%)
Unknown	293 (17%)	63 (21%)	60 (21%)
**Histological classification**			
Endometrioid endometrial carcinoma	1,099 (63%)	148 (49%)	139 (49%)
Non-endometrioid endometrial carcinoma	642 (37%)	151 (51%)	143 (51%)
**Lymph node**			
No regional lymph node involvement	1,021 (59%)	150 (50%)	140 (50%)
Regional lymph node involvement	720 (41%)	149 (50%)	142 (50%)
**Bone-metastases**			
No	926 (53%)	140 (47%)	129 (46%)
Yes	72 (4.1%)	29 (9.7%)	29 (10%)
Unknown	743 (43%)	130 (43%)	124 (44%)
**Brain-metastases**			
No	975 (56%)	160 (54%)	150 (53%)
Yes	23 (1.3%)	8 (2.7%)	8 (2.8%)
Unknown	743 (43%)	131 (44%)	124 (44%)
**Liver-metastases**			
No	915 (53%)	129 (43%)	120 (43%)
Yes	84 (4.8%)	44 (15%)	42 (15%)
Unknown	742 (43%)	126 (42%)	120 (43%)
**Lung-metastases**			
No	785 (45%)	93 (31%)	86 (30%)
Yes	214 (12%)	77 (26%)	73 (26%)
Unknown	742 (43%)	129 (43%)	123 (44%)
**Surgery**			
No	167 (9.6%)	128 (43%)	122 (43%)
Yes	1,574 (90%)	171 (57%)	160 (57%)
**Radiation**			
No	1,308 (75%)	279 (93%)	263 (93%)
Yes	433 (25%)	20 (6.7%)	19 (6.7%)
**Chemotherapy**			
No	433 (25%)	233 (78%)	221 (78%)
Yes	1,308 (75%)	66 (22%)	61 (22%)

Statistics presented: n (%); *American Indian/AK Native, Asian/Pacific Islander.

### Risk Factor Analysis for Early Death

Univariate and multivariate logistic regressions for all-cause and cancer-specific early death are shown in **Tables 2** and **3**. Univariate analysis showed that older patients; unmarried patients with large tumors; patients with high histological tumor grades, non-endometrioid endometrial carcinoma, regional lymph node metastasis, and bone, liver, or lung metastasis; and patients who did not receive surgery, radiation, or chemotherapy had a higher risk of all-cause early death. In addition to the above risk factors, univariate analysis showed that lack of insurance was also a risk factor for cancer-specific early death. Multivariate analysis showed that patients with large tumors (≥106 mm), high histological tumor grades (GIII-GIV), non-endometrioid endometrial carcinoma, and liver or lung metastases and those who did not undergo surgery, radiotherapy, or chemotherapy had a higher risk of all-cause and cancer-specific early death.

**Table 2:** The univariable logistic regression analysis of all-cause and cancer-specific early death.

Characteristic	All-cause early death			Cancer-specific early death		
	OR	95% CI	P-value	OR	95% CI	P-value
**Age**						
≤55	Reference			Reference		
56–74	1.76	1.26, 2.49	0.001*	1.64	1.17, 2.33	0.005*
≥75	3.28	2.25, 4.84	<0.001*	3.06	2.09, 4.52	<0.001*
**Race**						
White patients	Reference			Reference		
African American	1.10	0.75, 1.56	0.618	1.01	0.68, 1.46	0.967
Others**	1.11	0.74, 1.62	0.597	1.10	0.73, 1.61	0.651
**Insurance**						
Uninsured	Reference			Reference		
Insured	0.60	0.35, 1.10	0.080	0.56	0.33, 1.03	0.048*
Unknown	0.81	0.45, 1.53	0.502	0.78	0.43, 1.47	0.417
**Marital status**						
Unmarried	Reference			Reference		
Married	0.89	0.64, 1.24	0.482	0.91	0.65, 1.29	0.602
Divorced or Separated	1.04	0.66, 1.61	0.873	1.13	0.71, 1.77	0.601
Widowed	1.95	1.36, 2.81	<0.001*	1.97	1.36, 2.88	<0.001*
Unknown	0.76	0.32, 1.58	0.492	0.83	0.35, 1.72	0.636
**Tumor size(mm)**						
≤59	Reference			Reference		
60-105	1.55	1.17, 2.07	0.002*	1.46	1.09, 1.96	0.011*
≥106	2.88	2.06, 4.02	<0.001*	2.75	1.96, 3.86	<0.001*
**Histology grade**						
GI-II	Reference			Reference		
GIII-IV	2.68	1.89, 3.91	<0.001*	2.85	1.97, 4.23	<0.001*
Unknown	2.78	1.82, 4.30	<0.001*	2.96	1.91, 4.66	<0.001*
**Histological classification**						
Endometrioid endometrial carcinoma	Reference			Reference		
Non-endometrioid endometrial carcinoma	1.75	1.36, 2.24	<0.001*	1.76	1.37, 2.27	<0.001*
**Lymph node**						
No regional lymph node involvement	Reference			Reference		
Regional lymph node involvement	1.41	1.10, 1.80	0.006*	1.44	1.12, 1.85	0.005*
**Bone-metastases**						
No	Reference			Reference		
Yes	2.66	1.65, 4.21	<0.001*	2.89	1.79, 4.58	<0.001*
Unknown	1.16	0.89, 1.50	0.266	1.20	0.92, 1.56	0.181
**Brain-metastases**						
No	Reference			Reference		
Yes	2.12	0.88, 4.63	0.073	2.26	0.93, 4.95	0.052
Unknown	1.07	0.84, 1.38	0.574	1.08	0.84, 1.40	0.534
**Liver-metastases**						
No	Reference			Reference		
Yes	3.72	2.46, 5.57	<0.001*	3.81	2.50, 5.76	<0.001*
Unknown	1.20	0.92, 1.57	0.167	1.23	0.94, 1.62	0.129
**Lung-metastases**						
No	Reference			Reference		
Yes	3.04	2.16, 4.26	<0.001*	3.11	2.20, 4.40	<0.001*
Unknown	1.47	1.11, 1.96	0.008	1.51	1.13, 2.03	0.006*
**Surgery**						
No	Reference			Reference		
Yes	0.14	0.11, 0.19	<0.001*	0.14	0.10, 0.18	<0.001*
**Radiation**						
No	Reference			Reference		
Yes	0.22	0.13, 0.34	<0.001*	0.22	0.13, 0.34	<0.001*
**Chemotherapy**						
No	Reference			Reference		
Yes	0.09	0.07, 0.13	<0.001*	0.09	0.07, 0.12	<0.001*

OR, odds ratio; CI, confidence interval; *p < 0.05; ** :American Indian/AK Native, Asian/Pacific Islander.

**Table 3:** The multivariate logistic regression analysis of all-cause and cancer-specific early death.

Characteristic	All-cause early death			Cancer-specific early death		
	OR	95% CI	P-value	OR	95% CI	P-value
**Age**						
≤55	Reference			Reference		
56–74	1.19	0.80, 1.81	0.395	1.15	0.76, 1.75	0.522
≥75	1.27	0.77, 2.11	0.353	1.17	0.70, 1.97	0.549
**Insurance**						
Uninsured	–			Reference		
Insured	–	–	–	0.66	0.32, 1.42	0.274
Unknown	–	–	–	1.05	0.46, 2.47	0.906
**Marital status**						
Unmarried	Reference			Reference		
Married	0.87	0.58, 1.32	0.509	0.89	0.58, 1.37	0.595
Divorced or Separated	0.66	0.38, 1.14	0.142	0.71	0.41, 1.24	0.238
Widowed	1.08	0.66, 1.76	0.754	1.07	0.65, 1.77	0.791
Unknown	0.73	0.27, 1.76	0.506	0.71	0.26, 1.75	0.476
**Tumor size(mm)**						
≤59	Reference			Reference		
60–105	1.40	0.99, 1.98	0.057	1.29	0.91, 1.84	0.157
≥106	2.05	1.34, 3.13	<0.001*	1.94	1.25, 3.00	0.003*
**Histology grade**						
GI-II	Reference			Reference		
GIII-IV	3.22	2.09, 5.09	<0.001*	3.26	2.09, 5.24	<0.001*
Unknown	2.02	1.19, 3.47	0.010*	2.14	1.23, 3.74	0.007*
**Histological classification**						
Endometrioid endometrial carcinoma	Reference			Reference		
Non-endometrioid endometrial carcinoma	1.55	1.13, 2.12	0.006*	1.59	1.16, 2.20	0.005*
**Lymph node**						
No regional lymph node involvement	Reference			Reference		
Regional lymph node involvement	1.30	0.95, 1.78	0.096	1.32	0.96, 1.82	0.085
**Bone-metastases**						
No	Reference			Reference		
Yes	1.04	0.55, 1.92	0.896	1.15	0.61, 2.13	0.666
Unknown	44.1	3.47, 484	0.002*	50.3	3.92, 552	0.001*
**Liver-metastases**						
No	Reference			Reference		
Yes	2.09	1.22, 3.55	0.007*	2.14	1.24, 3.68	0.006*
Unknown	0.01	0.00, 0.18	0.002*	0.01	0.00, 0.14	<0.001*
**Lung-metastases**						
No	Reference			Reference		
Yes	2.23	1.44, 3.45	<0.001*	2.19	1.40, 3.43	<0.001*
Unknown	3.82	0.65, 21.6	0.136	3.90	0.62, 22.9	0.144
**Surgery**						
No	Reference			Reference		
Yes	0.20	0.14, 0.29	<0.001*	0.20	0.14, 0.29	<0.001*
**Radiation**						
No	Reference			Reference		
Yes	0.35	0.20, 0.58	<0.001*	0.34	0.19, 0.59	<0.001*
**Chemotherapy**						
No	Reference			Reference		
Yes	0.09	0.06, 0.12	<0.001*	0.09	0.06, 0.12	<0.001*

OR, Odds Ratio; CI, Confidence Interval, *p < 0.05.

### Nomogram Construction

**Figure 3:**
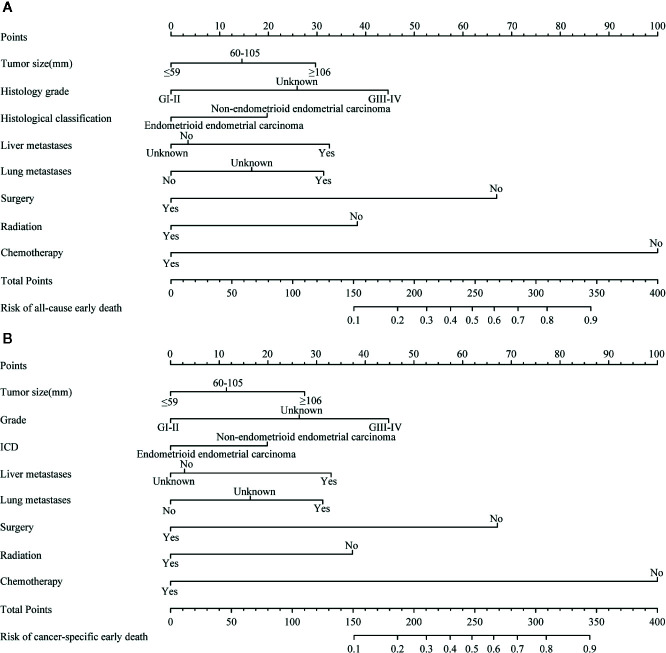
The nomograms of early death in patients with stage IV endometrial carcinoma. **(A)** |The all-cause early death. **(B)** The cancer-specific early death.

A nomogram of all-cause and cancer-specific early death was constructed by selecting important variables from the multiple logistic regression, including tumor size, histological grade, presence of metastasis (liver and/or lung), and treatment (surgery, radiotherapy, chemotherapy) (**Figure 3**).

### Performance of Nomograms

**Figure 4:**
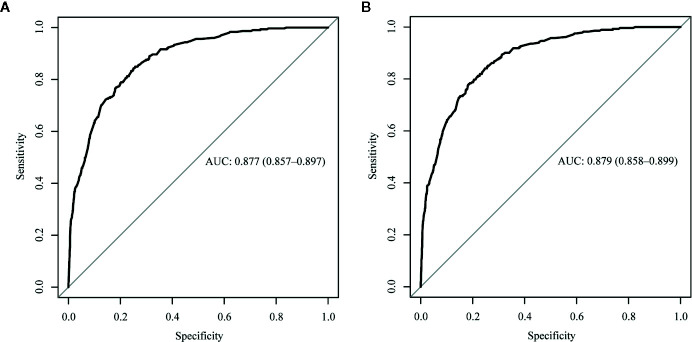
The receiver operating characteristic (ROC) curve for nomogram. **(A)** The all-cause early death. **(B)** The cancer-specific early death. AUC, area under the curve; ROC, receiver operating characteristic.

**Figure 5:**
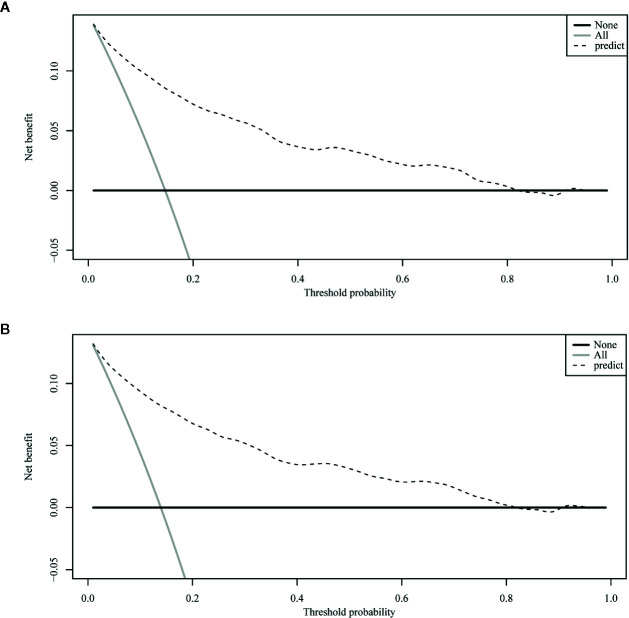
The decision curve analysis (DCA) curve for nomogram. **(A)** The all-cause early death. **(B)** The cancer-specific early death.

**Figure 6:**
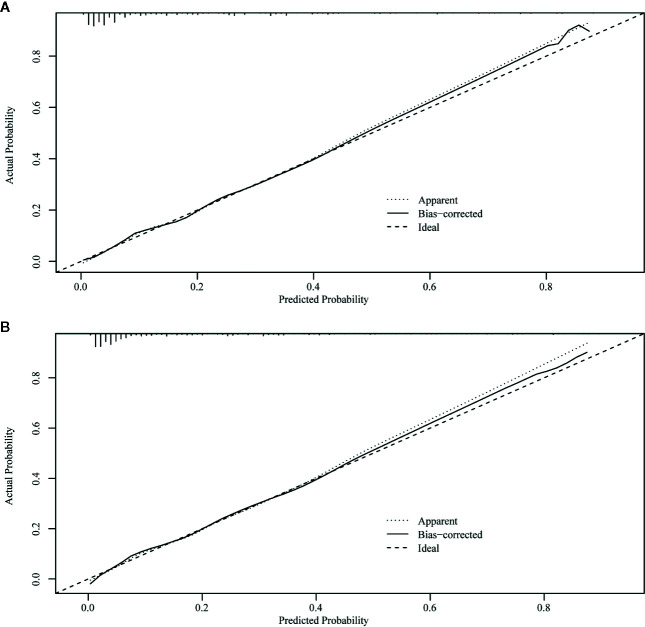
Internal verification plots of nomogram calibration curves by k-fold cross-validation (k =10). **(A)** The all-cause early death. **(B)** The cancer-specific early death.

The ROC curve of the nomograms used to evaluate all-cause and cancer-specific early death is shown in **Figure 4**. The AUC of the nomograms was higher than 85%, suggesting that the nomograms had a reliable prediction score for all-cause and cancer-specific early death. In addition, DCA results (**Figure 5**) showed a good clinical benefit for the management of endometrial carcinoma. Through internal verification, all calibration curves were observed to be close to the 45° line (**Figure 6**). The C statistics and Brier score before and after k-fold cross-validation (K =10) are shown in **Table 4**. An internal validation of the nomograms showed good consistency in the predicted values.

**Table 4:** C-statistic and Breir score of nomograms for the stage IV endometrial carcinoma.

Characteristics	Nomogram	After internal verification
**C-statistic**		
All-cause early death	0.8772	0.8673
Cancer-specific early death	0.8786	0.8713
**Brier score**		
All-cause early death	0.0854	0.0887
Cancer-specific early death	0.0820	0.846

## Discussion

Studies on the prognosis of malignant tumors usually focus on the long-term survival of patients (26, 27); only a few studies have focused on early death (19, 28). The risk of early death is higher for more aggressive or advanced tumors. The definition of early death varies from study to study (19, 29). Some studies defined it as 30 days or 3 months after surgery. Different definitions of the time of early death correspond to different early mortality rates. If the definition of early death is too short, the number of patients in the early death cohort is too small and the study bias is large. Finally, our study defined early death as 3 months after surgery. In this study, the early mortality rate from all causes for stage IV endometrial carcinoma was 14.7%, and the cancer-specific early mortality rate was 13.8%. Identifying patients at risk of early death is critical for reducing the burden on patients, for whom the side effects of treatment and the inconvenience of getting to the hospital may outweigh the benefits of treatment. Therefore, we constructed a nomogram for prediction of early death in patients with stage IV endometrial carcinoma. Demographic information such as age, race, and marital status is considered to be closely related to the prognosis of endometrial cancer (30–32). Our model analyzed the influence of such demographic factors on early death from stage IV endometrial cancer, but the results showed that they did not affect this outcome. Early death from stage IV endometrial carcinoma was mainly associated with clinical factors such as tumor size, histological stage, histological classification, liver and lung metastasis, and treatment (surgery, radiotherapy, and chemotherapy).

Our nomogram is based on the SEER database and thus was developed using a large sample size, which ensures the reliability and stability of the results. Our nomogram’s curve analysis and internal validation show that it has good discriminant and calibration abilities. With Nomogram, we can effectively screen out patients who are likely to die at an early stage, providing reference for subsequent individualized treatment of patients.

DCA is very useful in determining whether model-based clinical decisions are effective, while traditional ROC curve analysis is a statistical abstraction and cannot directly provide information about clinical value (33). Clinical practicality is an important indicator to determine whether predictive models can be used in clinical activities and whether patients can benefit from them; however, few studies have used this new method to assess the net benefits of forecasting models, and fewer still have applied it to predictive models for diseases of the endometrium. Zhu et al. (13) assessed the net benefits of their predictive model to estimate both overall and tumor-specific survival rates for endometrial carcinoma and found that it had clinical applicability. Our study also used a DCA curve to assess the clinical practicality of our model. In our prediction model, the nomogram’s net benefit was better than that in all-patient-death or no-patient-death scenarios at a threshold probability between 0% and 90%.

Some limitations of our study need to be mentioned. First, there were potentially relevant factors that were not included in the model. Estrogen and progesterone receptor expression, for example, is thought to be associated with endometrial cancer prognosis (34). Furthermore, familial endometrial carcinoma is associated with Lynch syndrome (35), which is an autosomal dominant susceptibility that is not included in the SEER database. Second, the SEER database does not have detailed information on chemotherapy, such as the use of hormone therapy (36), which is thought to ease the progression of advanced endometrial cancer. Third, the study did not consider specific surgical procedures, or radiotherapy regimens that may influence the prediction of early death. Fourth, at the beginning of the study, I tried to classify the histological subtypes of endometrial carcinoma in more detail, but due to the small number of patients included in each subtype, there was a large statistical bias. Therefore, I finally divided endometrial carcinoma into endometrioid endometrial carcinoma and non-endometrioid endometrial carcinoma for analysis without further classification. This may affect the results of the model. In addition, although the model was validated internally, we lacked external data to validate the model. In the future, the early death of stage IV endometrial carcinoma patients needs to be predicted in combination with other research data.

In summary, we identified factors associated with early mortality from endometrial carcinoma and developed a clinically useful nomogram to predict early mortality from stage IV endometrial carcinoma using a large cohort. This nomogram may help clinicians screen high-risk patients and administer individualized treatment regimens, and may improve survival outcomes of patients with stage IV endometrial carcinoma.

## Data Availability Statement

Publicly available datasets were analyzed in this study. This data can be found here: https://seer.cancer.gov/data/.

## Author Contributions

ZS provided research ideas and contributed to drafting. YW extracted the data and performed the statistical analysis. YZ performed the statistical analysis and wrote the manuscript. DZ conceptualized the research and conducted quality control. All authors contributed to the article and approved the submitted version.

## Conflict of Interest

The authors declare that the research was conducted in the absence of any commercial or financial relationships that could be construed as a potential conflict of interest.
